# CD169 Defines Activated CD14^+^ Monocytes With Enhanced CD8^+^ T Cell Activation Capacity

**DOI:** 10.3389/fimmu.2021.697840

**Published:** 2021-07-28

**Authors:** Alsya J. Affandi, Katarzyna Olesek, Joanna Grabowska, Maarten K. Nijen Twilhaar, Ernesto Rodríguez, Anno Saris, Eline S. Zwart, Esther J. Nossent, Hakan Kalay, Michael de Kok, Geert Kazemier, Johannes Stöckl, Alfons J. M. van den Eertwegh, Tanja D. de Gruijl, Juan J. Garcia-Vallejo, Gert Storm, Yvette van Kooyk, Joke M. M. den Haan

**Affiliations:** ^1^Department of Molecular Cell Biology and Immunology, Cancer Center Amsterdam, Amsterdam Institute for Infection and Immunity, Amsterdam UMC, Vrije Universiteit Amsterdam, Amsterdam, Netherlands; ^2^Center for Experimental and Molecular Medicine, Amsterdam UMC, Academic Medical Center, University of Amsterdam, Amsterdam, Netherlands; ^3^Department of Infectious Diseases, Leiden University Medical Center, Leiden, Netherlands; ^4^Department of Surgery, Cancer Center Amsterdam, Amsterdam UMC, Vrije Universiteit Amsterdam, Amsterdam, Netherlands; ^5^Department of Pulmonary Medicine, Amsterdam UMC, Vrije Universiteit Amsterdam, Amsterdam, Netherlands; ^6^Amsterdam Cardiovascular Sciences Research Institute, Amsterdam UMC, Amsterdam, Netherlands; ^7^Institute of Immunology, Centre for Pathophysiology, Infectiology and Immunology, Medical University of Vienna, Vienna, Austria; ^8^Department of Medical Oncology, Cancer Center Amsterdam, Amsterdam UMC, Vrije Universiteit Amsterdam, Amsterdam, Netherlands; ^9^Department of Pharmaceutics, Utrecht Institute for Pharmaceutical Sciences, Utrecht University, Utrecht, Netherlands; ^10^Department of Biomaterials, Science and Technology, Faculty of Science and Technology, University of Twente, Enschede, Netherlands; ^11^Department of Surgery, Yong Loo Lin School of Medicine, National University of Singapore, Singapore, Singapore

**Keywords:** monocyte, CD169, antigen-presentation, CD8^+^ T cell, nanovaccine, cancer, COVID-19, Siglec-1

## Abstract

Monocytes are antigen-presenting cells (APCs) that play diverse roles in promoting or regulating inflammatory responses, but their role in T cell stimulation is not well defined. In inflammatory conditions, monocytes frequently show increased expression of CD169/Siglec-1, a type-I interferon (IFN-I)-regulated protein. However, little is known about the phenotype and function of these CD169^+^ monocytes. Here, we have investigated the phenotype of human CD169^+^ monocytes in different diseases, their capacity to activate CD8^+^ T cells, and the potential for a targeted-vaccination approach. Using spectral flow cytometry, we detected CD169 expression by CD14^+^ CD16^-^ classical and CD14^+^ CD16^+^ intermediate monocytes and unbiased analysis showed that they were distinct from dendritic cells, including the recently described CD14-expressing DC3. CD169^+^ monocytes expressed higher levels of co-stimulatory and HLA molecules, suggesting an increased activation state. IFNα treatment highly upregulated CD169 expression on CD14^+^ monocytes and boosted their capacity to cross-present antigen to CD8^+^ T cells. Furthermore, we observed CD169^+^ monocytes in virally-infected patients, including in the blood and bronchoalveolar lavage fluid of COVID-19 patients, as well as in the blood of patients with different types of cancers. Finally, we evaluated two CD169-targeting nanovaccine platforms, antibody-based and liposome-based, and we showed that CD169^+^ monocytes efficiently presented tumor-associated peptides gp100 and WT1 to antigen-specific CD8^+^ T cells. In conclusion, our data indicate that CD169^+^ monocytes are activated monocytes with enhanced CD8^+^ T cell stimulatory capacity and that they emerge as an interesting target in nanovaccine strategies, because of their presence in health and different diseases.

## Introduction

Monocytes are members of the innate immune system circulating in the blood that are important in sensing danger signals such as pathogens. They comprise of about 10-15% of human peripheral blood mononuclear cells (PBMCs) and their many roles include phagocytosis of pathogens or foreign bodies, differentiation into tissue macrophages upon inflammation, and antigen presentation. Together with dendritic cells (DCs) they function as antigen-presenting cells (APCs) that activate the adaptive immune responses, including CD4^+^ and CD8^+^ T cells (1). Furthermore, their potency in the production of pro- and anti-inflammatory cytokines allows them to govern both local and systemic immunity.

Monocytes can be broadly categorized into classical (CD14^+^ CD16^-^), intermediate (CD14^+^ CD16^+^), and non-classical (CD14^-^ CD16^+^) populations (2). Monocytes originate in the bone marrow, then they enter the blood stream as classical monocytes, and further differentiate into intermediate monocytes and to non-classical monocytes in a linear fashion (3, 4). While non-classical monocytes have a ‘patrolling function’, crawling the endothelium and supporting blood vessels integrity, classical monocytes have the ability to enter tissue or lymphoid organs. Depending on the tissue microenvironmental cues, tissue-migrated monocytes have diverse differentiation potential, giving rise to tissue macrophages with inflammatory or resolving functions (5).

During inflammation, monocytes can also acquire DC-like properties; however, their precise characterization, definition, and functions are continuously evolving (1, 6). *In vitro*, monocytes exposed to GM-CSF and IL-4 (moDC) are able to prime and to stimulate T cell responses. Although they are distinct from naturally occurring DCs found in the circulation, *ex vivo* generated moDCs have been used during initial efforts in the development of cancer vaccination. However, moDC-based vaccination is laborious and costly, hence more research has focused on targeting of naturally occurring APCs *in situ* (7, 8).

The distribution and numbers of monocyte subsets can change dramatically under inflammatory conditions, such as during bacterial or virus infection. In COVID-19 patients, HLA-DR^hi^ inflammatory monocytes were reported to be increased in patients with mild symptoms, whereas HLA-DR^lo^ monocytes were more prominent in severely ill patients (9). While these monocytes also expressed high co-stimulatory molecules and pro-inflammatory cytokines (9, 10), their contribution towards activation of virus-specific T cell responses remains unclear.

In cancer, monocytes are thought to contribute to tumor progression as the major source of tumor-associated macrophages or myeloid-derived suppressor cells with high immune-suppressive activity (11). However, monocytes have also anti-tumoral roles; they can engulf tumor cells and process them for antigen presentation and they elicit direct tumoricidal activities (11–14). Moreover, high frequency of circulating classical monocytes have been described to be predictive of a successful anti-PD1 immunotherapy in melanoma (15), suggesting that monocytes can play an important role in activating anti-tumor T cell responses in cancer.

With the rise of single-cell approaches, recent studies have broadened the heterogeneity of DC and monocyte subsets. Until recently, DCs were categorized into the type-I interferon (IFN-I) producing CD123^+^ plasmacytoid DCs (pDCs) and conventional DCs (cDCs) that include CD141^+^ DC1 and CD1c^+^ DC2, which both have high antigen-presentation and T cell-activating potential (16). Two single-cell RNA sequencing (scRNA-seq) studies have identified Axl^+^ DC (pre-DC/AS-DC) that expresses Axl, Siglec-6, and CD169 (17, 18). Axl^+^ DCs are unable to produce IFN-I, but they can present antigen and activate T cells and can further differentiate into DC1 or DC2 (17–20). Next to this, DC3 has also been identified as a new subset of DC that displays a monocyte/DC2 hybrid phenotype (21, 22). Since DC3 express classical monocytic markers such as CD14 and CD163, the inclusion of CD88 and FcϵRIa as markers have been used to better discriminate between monocyte and DC3, respectively (23, 24). DC3s are proficient in activating T cells and they were expanded in patients with systemic lupus erythematosus, melanoma, and breast cancer (23–25).

CD169 (Siglec-1, sialoadhesin) is a sialic-acid binding transmembrane receptor that is expressed mainly by a subset of macrophages in the spleen and lymph nodes (CD169^+^ macrophages). These macrophages function as gatekeeper of the immune system and the CD169 molecule is involved in pathogen capture and antigen transfer to DCs, leading to T cell activation (26). CD169 is also expressed by monocytes, moDC, and Axl^+^ DC, and its expression is upregulated by type I interferon (IFN-I). In inflammatory conditions where IFN-I levels are high, such as in autoimmunity or viral infection, CD169 expression in monocytes is increased (27–30). Moreover, viruses such as HIV are able to exploit CD169-sialic acid interaction, by incorporating ganglioside GM3 in their membrane, to infect CD169^+^ monocytes or DCs (31–33).

Based on the CD169-sialic acid interaction, we designed a lipid-based nanovaccine that can selectively target CD169^+^ APCs, by incorporating GM3 or other gangliosides in the liposome membrane (20, 34, 35). Using human cells, we showed that ganglioside-liposomes could stimulate tumor-antigen specific CD8^+^ T cell responses mediated by CD169 expression on Axl^+^ DCs. Next to Axl^+^ DCs, we also observed that CD169^+^ monocytes could efficiently bind ganglioside-liposomes, however, whether they contribute to CD8^+^ T cell activation is unknown.

In this study, we used spectral flow cytometry to perform immunophenotypical analyses of CD169^+^ monocytes as compared to DC subsets in PBMCs of healthy individuals. By combining spectral flow cytometry and analysis of public single-cell RNA sequencing datasets, we examined the presence of CD169^+^ monocytes in COVID-19 patients and patients with five different cancer types. We then determined the capacity of CD169^+^ monocytes to cross-present tumor-associated gp100 peptides to CD8^+^ T cells. Furthermore, we evaluated vaccination strategies that specifically target antigens to CD169^+^ monocytes using anti-CD169 antibodies and ganglioside-liposomes. Here, we demonstrate that CD169 expression reflect a higher activation status of monocytes, with an enhanced CD8^+^ T cells activating capacity. Importantly, delivery of tumor-antigen to CD169^+^ monocytes using two forms of CD169-targeting nanovaccines leads to robust activation of antigen-specific CD8^+^ T cell responses.

## Materials and Methods

### Study Patients

Human peripheral blood mononuclear cells (PBMC) were collected from patients with gastrointestinal malignancies, metastatic melanoma, or COVID-19 in accordance with the Helsinki Declaration of 1975 and approved by the institutional ethical review board of the Amsterdam Universitair Medische Centra (UMC). All patients provided written consent for research purposes. Informed consent was deferred until discharge from the intensive care units (ICU). In case of death, informed consent was requested from the patient’s relatives. COVID-19 patients were enrolled in the ArtDECO-1 study, a cohort study of COVID-19 patients with persistent acute respiratory distress syndrome (ARDS). Leftover biological samples were stored in the anonymized Amsterdam UMC COVID-19 biobank (#2020-182). This study procedure was approved by the Review Committee Biobank of the Amsterdam UMC (2020-065).

Pancreatic ductal adenocarcinoma (PDAC), hepatocellular carcinoma (HCC), and colorectal liver metastasis (CRLM) patients were enrolled in the Hepatobiliary (HPB) biobank at Amsterdam UMC, location VU University medical center (VUmc, Medical Ethical Committee approval 2016.510). Melanoma patients were enrolled in a clinical study of autologous whole-cell vaccination at the VUmc between 1987 and 1998 (36). Leftover human spleen tissue was obtained anonymously from the VUmc Biobank (BUP 2015-074), therefore approval by the Medical Ethical Committee was not required.

### Isolation of Human Primary Cells

PBMCs from heparinized blood were isolated by density gradient centrifugation (Lymphoprep; Axis-Shield PoC AS). To isolate bronchoalveolar lavage fluid mononuclear cells (BALFMCs), during bronchoscopy lungs were instilled with 2 x 20 ml 0.9% NaCl for diagnostics and remaining 3-20 ml was centrifuged. Cell pellets were suspended in 2 mM dithiothreitol (Sigma), and BALFMCs were isolated using Ficoll isolation and cryopreserved. Human spleen was mechanically and enzymatically digested with Liberase and DNAse I (Roche) at 37°C for 30 min. Cells were then depleted of red blood cells using ammonium chloride lysing buffer. Following PBS washes, cells were further processed as described below.

### Monocyte Isolation and Culture

CD14^+^ monocytes were isolated using magnetic beads (Miltenyi) on LS column according to manufacturer’s recommendation. Where indicated, monocytes were isolated using Percoll density gradient. Monocytes were cultured in RPMI 1640 complete medium (Thermo Fisher Scientific) containing 10% fetal calf serum (Biowest), 50 U/mL penicillin, 50 μg/mL streptomycin, and 2 mM glutamine (all from Thermo Fisher Scientific). Monocytes were then treated with recombinant human IFNα (Miltenyi Biotec) at indicated doses and time-points.

### Antibody-Antigen (Ab-Ag) Conjugation

As described previously, gp100 [YLEPGPVTAC-6-ahx-lysine (biotin)] peptide was conjugated to purified anti-CD169 (clone 7-239, produced in house) or control mouse IgG, using sulfhydryl-based coupling (37). In short, purified antibodies (Abs) were functionalized with 2–8 equivalents of SMCC [succinimidyl 4-(N-maleimidomethyl) cyclohexane-1-carboxylate, Thermo Fisher Scientific]. After purification over PD-10 columns (GE Life Sciences) activated Abs were concentrated with centricon 30 (Merck Millipore) to 500 µL. 2–4 Equivalents of peptides in 50 µl was added to the Abs and after 1 h incubation at room temperature conjugates were purified over a Sephadex 75 10/30 column (GE Life Sciences) according to manufacturer’s HPLC settings. Concentration was determined using BCA assay (Pierce, Thermo Fisher Scientific).

### Liposome Preparation

Liposomes were prepared from a mixture of phospholipids and cholesterol utilizing the film extrusion method as described previously (38, 39). In brief, egg phosphatidylcholine (EPC)-35 (Lipoid GmbH): egg phosphatidylglycerol (EPG)-Na (Lipoid GmbH): Cholesterol (Sigma-Aldrich) were mixed at a molar ratio of 3.8:1:2.5. 3 mol% of ganglioside (GM3, Avanti Polar Lipids; GT1b, Matreya LLC), and 0.1 mol% of lipophilic fluorescent tracer DiD (1,1′-dioctadecyl-3,3,3′,3′-Tetramethylindodicarbocyanine, Thermo Fisher Scientific). TLR-ligand R848 (4 mol%, Invivogen) was included where specified. The solvent was evaporated under vacuum on a rotavapor to generate a lipid film and the residual organic solvent was removed by nitrogen flush. The lipid film was then hydrated in HEPES-buffered saline (10 mM HEPES buffer pH 7.4, 0.8% NaCl) with mechanical agitation by rotary-mixing for 20 min until the lipid film was completely resuspended. For antigen presentation assay, the pancreatic cancer-associated antigen Wilms’ Tumor 1 (WT1) short peptide (RMFPNAPYL, 3 mg/ml) was encapsulated into the liposomes during the hydration step. Peptides were produced by solid phase peptide synthesis using Fmoc-chemistry with a Symphony peptide synthesizer (Protein Technologies Inc). The liposomes were sized by sequential extrusion through two stacked polycarbonate filters (400 and 200 nm) with Lipex high-pressure extrusion device (Northern Lipids). Non-incorporated materials were removed in two consecutive steps by sedimentation of the liposomes by ultracentrifugation at 200,000 x g. The final resuspension of the liposomes was performed in HEPES buffer at pH 7.4. The mean particle size, polydispersity index, and zeta potential were measured using Malvern Zetasizer (Malvern Instruments). Physical properties of liposomes are shown in **Table S1**.

### Liposome Uptake

Cells were incubated with ganglioside-liposomes (100 µM, unless indicated otherwise) for 45 min at 37°C to evaluate liposomes uptake. Specific uptake of ganglioside-liposomes mediated by CD169 was determined by pre-incubation of cells for at least 15 min at 4°C with 2 µg/ml neutralizing antibody against CD169, clone 7-239.

### Flow Cytometry

Cells were incubated with Fc block (BD Biosciences, cat. #564219) and viability dye (Fixable viability dye eFluor 780 or 455UV, FVD, eBioscience), prior to cell surface staining with fluorescence-conjugated antibodies in 0.5% BSA/PBS for 20 min at 4°C. After thorough washes, cells were fixed with 2% paraformaldehyde for 10 min at RT. For intracellular staining, cells were additionally incubated with antibodies in 0.5% BSA/PBS with 0.5% saponin for 20 min at 4°C. Cells were acquired on Fortessa (BD Biosciences) or Aurora spectral flow cytometer (Cytek) and analyzed on FlowJo software (Tree Star) or OMIQ. High dimensionality reduction analysis opt-SNE was performed using FlowJo software. Antibody clones and dilutions used are listed in **Table S2**. Antibodies and reagents used for the ArtDECO cohort have been listed elsewhere (40). BALF samples were excluded when too few viable CD45^+^ cells were measured (i.e., less than 2,000) or during active therapy with corticosteroids, as described previously (40). PBMC samples were only included if a paired BALF sample was available.

### Monocyte Activation and Antigen Presentation

PBMCs were incubated with ganglioside-liposomes at 37°C for 45 min, washed, and cultured for 5 hours in RPMI complete medium, with the addition of Brefeldin A (BD GolgiPlug) for the final 3 hours. TNFα production was measured by intracellular flow cytometry. For antigen presentation, CD14^+^ monocytes were incubated with gp100 synthetic peptides (short, YLEPGPVTA; long, VTHTYLEPGPVTANRQLYPEWTEAQRLD; 3h, 37°C), ganglioside-liposomes encapsulating WT1 short peptide (1h, 37°C), or antibody-gp100 conjugates (30 min, 4°C), followed by medium washes. R848 (2.5 µg/ml, Invitrogen) was added to the soluble peptides or Ab-Ag conjugates conditions during uptake. Antigen-loaded monocytes were then co-cultured overnight with WT1_126-134_ or gp100_280–288_ T-cell receptor (TCR) transduced HLA-A2.1 restricted T cell lines (0.5-1 × 10^5^ cells per well), at a ratio of 1:1. After 24 h, production of IFNγ in the supernatants of the co-cultures was determined by ELISA (eBioscience).

### Analysis of Single-Cell RNA Sequencing

We performed analysis of public datasets from patients with PDAC (GSE155698), lung cancer (GSE127465), as well as patients with severe influenza or COVID-19 (SARS-CoV-2) and healthy donors (GSE149689). We used Seurat package (version 3.2.2) and unsupervised UMAP high dimensionality reduction analysis, with R version 4.0.3. IFN-I score (GSEA GO:0034340) and TLR activation (KEGG hsa04620) score were calculated using AddModuleScore function. Codes are available upon request.

### Statistics

Statistical analysis of Friedman test, corrected using a two-stage linear step-up procedure of Benjamini, Krieger and Yekutieli, or paired t-test, were performed using GraphPad Prism 8 (GraphPad Software), unless indicated otherwise.

## Results

### CD169 Is Expressed by CD14^+^ Monocytes With Increased Maturation Status in Healthy Donors

**Figure 1:**
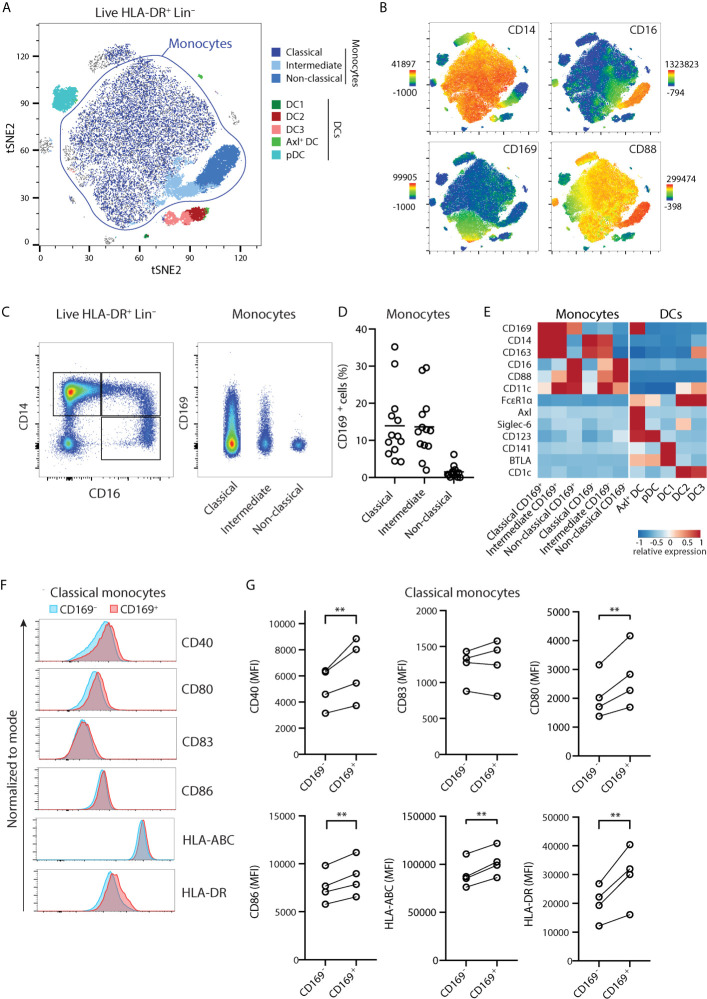
CD14^+^ CD169^+^ monocytes display enhanced maturation status. **(A)** Unsupervised high dimensionality reduction analysis of circulating monocytes and dendritic cells (HLA-DR^+^ CD3/CD19/CD56^–^) using CD14, CD16, CD88, CD163, HLA-DR, FcϵR1α, CD123, CD11c, CD1c, CD141, Axl, Siglec-6, BTLA, and CD169 using opt-SNE, and overlaid with conventional gating. **(B)** The expression of CD14, CD16, CD88, and CD169 on tSNE plots. **(C)** Gating strategy identifies classical (CD14^+^ CD16^-^), intermediate (CD14^+^ CD16^+^), or non-classical (CD14^-^ CD16^+^) monocytes, and CD169^+^ cells. **(D)** Percentage of CD169^+^ cells within monocytes subsets (*n* = 13) in healthy donors. **(E)** Heatmaps comparing the relative expression of markers defining monocytes and DC subsets. **(F, G)** Expression of co-stimulatory and HLA molecules are compared between CD169^+^ and CD169^-^ classical monocytes shown as **(F)** representative histograms and **(G)** quantification (*n* = 4). Paired t-tests were used. **P < 0.01.

We previously showed CD169 expression within HLA-DR^+^ CD14^+^ Lin^-^ populations (20). However, CD14 expression is not restricted to monocytes and can also be observed in CD1c^+^ DC3 (17, 23). We then sought a strategy to define monocytes and DC subsets unequivocally based on markers identified recently (23, 24), by including CD88 to identify *bona fide* monocytes, and FcϵRIα to distinguish the DC lineage. We performed unsupervised spectral cytometry analysis of circulating HLA-DR^+^ Lin^-^ cells from healthy donors using opt-SNE (**Figure 1A**) overlaid with conventional gating (**Figure S1A**). By using CD88 to classify total monocytes, we identified classical (CD14^+^ CD16^-^), intermediate (CD14^+^ CD16^+^), and non-classical (CD14^-^ CD16^+^) monocytes. The CD88^-^ DC populations consisted of Axl^+^ DC (Axl^+^ Siglec-6^+^), pDC (CD123^+^), DC1 (CD141^+^), DC2 (CD1c^+^), and the CD14-expressing DC3 (CD1c^+^ CD163^+^). As we described previously, CD169 expression was found within CD14^+^ monocytes and Axl^+^ DCs (**Figures 1A-C**). A proportion of DC3 also expressed CD169, however they expressed FcϵRIα^+^ and lacked CD88, confirming their DC lineage (**Figure 1A** and **Figures S1B, C**). Thus, although both cell types share the expression of CD14 and other markers, CD14^+^ CD169^+^ monocytes are distinct from DC3.

Among monocytes, CD169^+^ cells were present within classical and intermediate monocytes, and they were much less frequent in non-classical monocytes (**Figures 1C–E**). When stratified based on CD169 expression, CD14^+^ CD169^+^ monocytes expressed a similar level of CD169 to Axl^+^ DCs (**Figure 1C** and **Figure S1C**). Since CD14^+^ CD169^+^ monocytes were frequently found in inflammatory conditions, we screened for expression of maturation markers. Interestingly, CD169^+^ classical monocytes expressed higher levels of co-stimulatory molecules CD40, CD80, CD86, HLA-ABC, and HLA-DR, as compared to CD169^-^ classical monocytes (**Figures 1F–G**). A similar increase of maturation markers was found in CD169-expressing intermediate monocytes (**Figure S1D**). These suggest that CD14-expressing classical and intermediate CD169^+^ monocytes represent monocytes with a higher activation status.

### CD169-Expressing CD14^+^ Monocytes in Viral Infection

**Figure 2:**
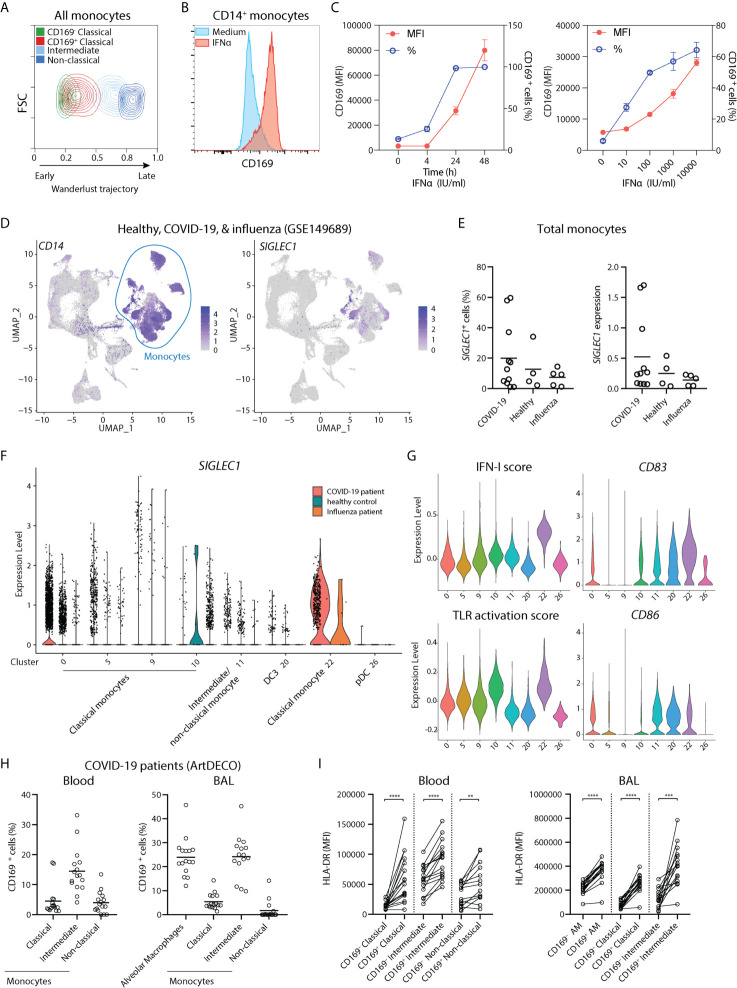
CD169 expression in monocytes is driven by IFN-I and CD14^+^ CD169^+^ monocytes are present in COVID-19 patients. **(A)** Wanderlust trajectory analysis of monocyte population using CD14^+^ CD169^-^ monocytes as starting population overlaid by conventional gating of monocyte subsets. **(B, C)** CD14^+^ monocytes were isolated and treated with 1,000 IU/ml IFNα for 24h unless indicated otherwise. Percentage of CD169^+^ cells or CD169 median fluorescence intensity (MFI) of CD169^+^ population are shown. (*n* = 4). **(D–G)** Analysis of public scRNA-seq dataset of PBMCs from patients with COVID-19 (*n* = 9), severe influenza (*n* = 5) and healthy controls (*n* = 4) using Seurat pipeline. **(D)** UMAP analysis showing the expression of *CD14* and *SIGLEC1*. **(E)** *SIGLEC1* expressing monocytes as shown as percentages or MFI in different groups. **(F)** Violin plot of *SIGLEC1* expression in clusters of monocytes and DC subsets from each patient group. **(G)** Violin plots of IFN-I score, TLR activation score, maturation markers *CD83* and *CD86*, in monocytes and DC clusters of all groups. **(H, I)** Spectral flow cytometry analysis of COVID-19 patients (ArtDECO cohort) of CD169-expressing monocytes/macrophages in circulation or bronchoalveolar space. **(H)** Percentage of CD169^+^ cells within monocytes subsets or alveolar macrophages (AM) (*n* = 16) in the (left panel) blood or (right panel) BALF of COVID-19 patients. **(I)** Expression of HLA-DR compared between CD169^+^ and CD169^-^ subsets of monocytes or alveolar macrophages (AM). Paired t-tests were used. **P < 0.01, ***P < 0.001, ****P < 0.0001.

CD169 expression in monocytes has been shown to be upregulated upon exposure to type I interferon (IFN-I) in many inflammatory conditions, including in autoimmune diseases and viral infections (27–30). To predict their developmental trajectories, we applied Wanderlust analysis using CD14^+^ CD169^-^ classical monocytes as a starting population and the inclusion of 9 phenotypic markers. CD14^+^ CD169^+^ monocytes were in close proximity to CD14^+^ CD169^-^ monocytes, whereas intermediate and non-classical monocytes were further away along the trajectory (**Figure 2A**). The expression of CD169 was also increased at an early stage (**Figures S2A, B**), suggesting that CD169-expressing monocytes arise immediately from CD14^+^ CD169^-^ classical monocytes. Next, we isolated CD14^+^ monocytes and exposed them to IFNα. In line with previous findings, we observed an increased expression of CD169 in a time- and dose-dependent manner (**Figures 2B, C** and **Figure S2C**). IFNα treatment led to upregulation of HLA-DR, CD80, CD86, and CD16, in CD14^+^ monocytes, although we noticed that CD16 expression was already high upon culture with medium alone (**Figures S2D–F**). CD169 expression was also increased in CD14^-^ CD16^+^ non-classical monocytes, albeit at a much lower extent (**Figures S2G, H**). Transcriptomic analyses of IFNβ-treated monocytes showed similar findings (**Figures S2I–P**). Thus, CD169 upregulation in circulating CD14^+^ monocytes is likely to be driven by increased IFN-I levels during inflammation.

Since IFN-I is a crucial component of host defense against viruses, we then analyzed a recently published single-cell RNA sequencing (scRNA-seq) dataset of PBMCs of patients with COVID-19, severe influenza, and healthy controls (41). We applied Seurat pipeline and subjected them to Uniform Manifold Approximation and Projection (UMAP) dimensionality reduction analysis. UMAP visualization identified all major immune cell populations, including clusters of classical monocytes as identified by *CD14* and *VCAN* (cluster 0, 5, 9, 10, and 22), and cells expressing *FCGR3A* (encoding CD16) and *MS4A7*, evoking intermediate/non-classical monocytes (cluster 11) (**Figure 2D** and **Figure S3**). Two DC populations were also found: the *FCER1A* ^+^ *CD1C* ^+^ DC3 (cluster 20), which expressed low *CD14* but lacked *C5AR1* (encoding CD88), and *LILRA4^+^ IL3RA^+^* pDCs (cluster 26) (**Figure S3**). *SIGLEC1* (encoding CD169) transcript was mainly detected within clusters of monocytes and appeared to be increased in a few COVID-19 patients (**Figures 2E, F**). Focusing on monocytes, we observed that *SIGLEC1* was highly increased in cluster 22, which was a cluster almost exclusively comprised of COVID-19 patients (**Figure 2F**). Remarkably, *SIGLEC1* was one of the most highly expressed genes in this cluster as compared to other monocytes (**Table S3**). Furthermore, cells in cluster 22 showed increased expression of genes involved in IFN-I pathway and TLR signaling (**Figure 2G**), and expressed high levels of *CD83* and *CD86*, indicating an increased activation state of monocytes. To confirm this on protein level, we examined CD169 expression in blood and BALF of COVID-19 patients of the ArtDECO cohort using spectral cytometry. Indeed, CD169^+^ monocytes were present in both blood and BALF of these patients (**Figure 2H** and **Figure S3F**). CD169 was also expressed in alveolar macrophages, as we described previously (40). The expression of HLA-DR was also consistently higher in CD169^+^ cells monocytes/macrophages (**Figure 2I**). These observations indicate that CD169^+^ monocytes are activated monocytes associated with IFN-I signature upon viral infection, and are present in the circulation and lungs of COVID-19 patients.

### CD14^+^ CD169^+^ Monocytes Are Present in Cancer Patients

**Figure 3:**
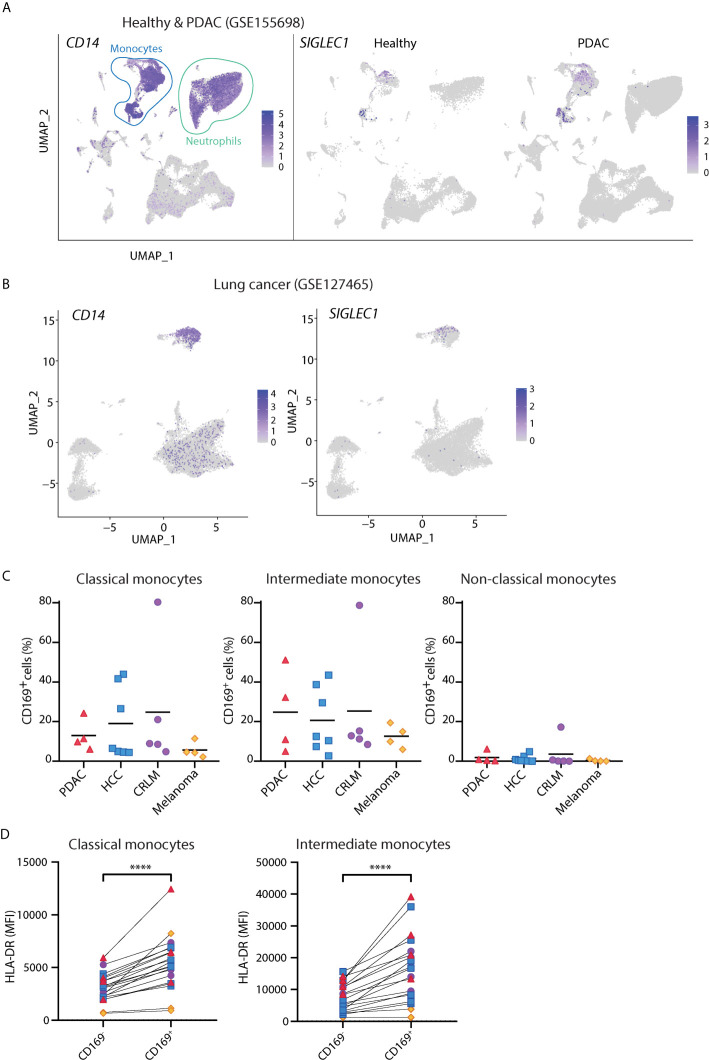
CD14^+^ CD169^+^ monocytes are present in cancer patients. **(A)** Analysis of public scRNA-seq dataset of PBMCs from PDAC patients (*n* = 12) and healthy controls (*n* = 4) using Seurat algorithm and projected onto UMAP space where cell types are indicated. The expressions of *CD14* and *SIGLEC1* are visualized on UMAP. **(B)** Analysis of public scRNA-seq dataset of PBMCs from patients with lung cancer (*n* = 7) using Seurat and UMAP clustering. The expression of *SIGLEC1* and *CD14* are shown. **(C)** Percentage of CD169^+^ cells within classical (CD14^+^ CD16^-^), intermediate (CD14^+^ CD16^+^), or non-classical (CD14^-^ CD16^+^) monocytes in patients with pancreatic ductal adenocarcinoma (PDAC, *n* = 4), hepatocellular carcinoma (HCC *n* = 7), colorectal liver metastasis (CRLM, *n* = 4), and melanoma (*n* = 4). Monocytes were gated on live, HLA-DR^+^ Lin(CD3/CD19/CD56)^–^ cells. **(D)** Expression of HLA-DR between CD169^+^ and CD169^-^ classical or intermediate monocytes in cancer patients. Paired t-tests were used. ****P < 0.0001.

Although the presence of CD169^+^ macrophages in draining lymph nodes has been associated with anti-tumor responses (42–44), there is little known about circulating CD169^+^ monocytes in cancer. We analyzed public scRNA-seq dataset of PBMCs from PDAC patients [GSE155698 (45)] and healthy controls. UMAP analysis showed that cells expressing *SIGLEC1* transcript were found primarily within clusters of *CD14*-expressing monocytes (**Figure 3A** and **Figure S4**), confirming our flow cytometry findings. Similar observations were made for the scRNA-seq dataset of PBMCs from lung cancer patients [GSE127465 (46), **Figure 3B**]. We further validated these findings in PBMCs of patients with PDAC, HCC, CRLM, and melanoma, using flow cytometry. Indeed, we were able to identify CD169^+^ cells among classical and intermediate monocytes and they expressed higher HLA-DR than the CD169^-^ counterparts (**Figures 3C, D**). Thus, CD169^+^ monocytes are present in the circulation of cancer patients.

### IFNα Treatment Gives Rise to CD14^+^ CD169^high^ Monocytes With an Enhanced CD8^+^ T Cell Activating Capacity

**Figure 4:**
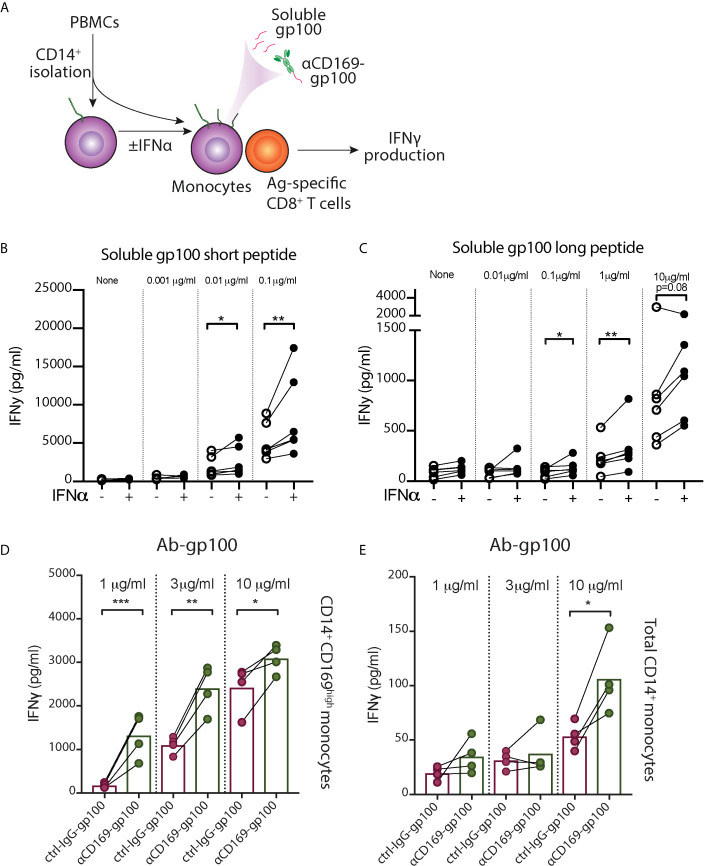
IFN-α treated monocytes showed enhanced peptide presentation to CD8^+^ T cells. **(A–C)** After CD14^+^ isolation, monocytes were incubated with IFNα overnight, loaded with different concentrations of gp100, washed, and gp100-specific CD8^+^ T cells were added. After 24h, IFNγ secretion by CD8^+^ T cells after co-culture with monocyte loaded with **(B)** short peptide or **(C)** long peptide was measured by ELISA. **(D, E)** Targeted antigen delivery to CD14^+^ CD169^+^ monocytes using antibody conjugated with gp100 peptide. **(D)** IFNα-treated CD14^+^ CD169^high^ monocytes or **(E)** freshly-isolated total CD14^+^ monocytes were loaded with different doses of αCD169-gp100 or control IgG-gp100 antibody-conjugates, washed, and gp100-specific CD8^+^ T cells were added. After 24h IFNγ secretion was measured by ELISA. Data are mean from four donors. Paired t-tests were used. *P < 0.05, **P < 0.01, ***P < 0.001.

The increased expression of activation markers in CD169^+^ monocytes propelled us to investigate their capacity to stimulate T cells. Since CD169 expression in monocytes is heterogenous, we used CD14^+^ monocytes exposed to IFNα, which showed high homogenous levels of CD169 (CD169^high^ monocytes), for functional analysis (**Figure S2**). To assess their antigen-presentation capacity, we incubated monocytes with pre-processed melanoma-associated peptides gp100 (short peptide), followed by co-culture with gp100_280–288_-specific CD8^+^ T cells (**Figure 4A**). We observed an increased IFNγ secretion by CD8^+^ T cells after co-culture with antigen-loaded CD169^high^ monocytes compared to untreated monocytes (**Figure 4B**). Subsequently, we assessed their antigen-processing and cross-presentation abilities using gp100 long peptide. Interestingly, CD169^high^ monocytes were able to stimulate a higher amount of IFNγ production by gp100-specific CD8^+^ T cells in almost every donor (**Figure 4C**), indicating their cross-presentation potential. Next to the increased expression of activation markers, these data suggest that IFNα-induced CD14^+^ CD169^+^ monocytes have an enhanced capacity to cross-present antigen and to stimulate CD8^+^ T cells.

### Targeted Antigen Delivery to CD14^+^ CD169^+^ Monocytes Using Ab-Ag Stimulate CD8^+^ T Cell Activation

To evaluate whether CD14^+^ CD169^+^ monocytes can be used for targeted vaccination, we conjugated gp100 peptide to αCD169 antibody. After incubation with αCD169-gp100 or control-IgG-gp100 conjugates, IFNα-treated CD169^high^ monocytes were washed and co-cultured with gp100_280–288_-specific CD8^+^ T cells. We found a significantly higher IFNγ secretion by CD8^+^ T cells in αCD169-gp100-treated condition (**Figure 4D**). Similar result was seen when we used freshly-isolated CD14^+^ monocytes, however, the level of IFNγ production was much lower due to a lower expression of CD169 on only a small percentage (5-15%) of total monocytes (**Figure 4E**). This suggests that CD14^+^ CD169^+^ monocytes can potentially be used for an effective targeted vaccination strategy.

### Ganglioside-Liposomes Target CD14^+^ CD169^+^ Monocytes Leading to Antigen-Presentation to CD8^+^ T Cells

**Figure 5:**
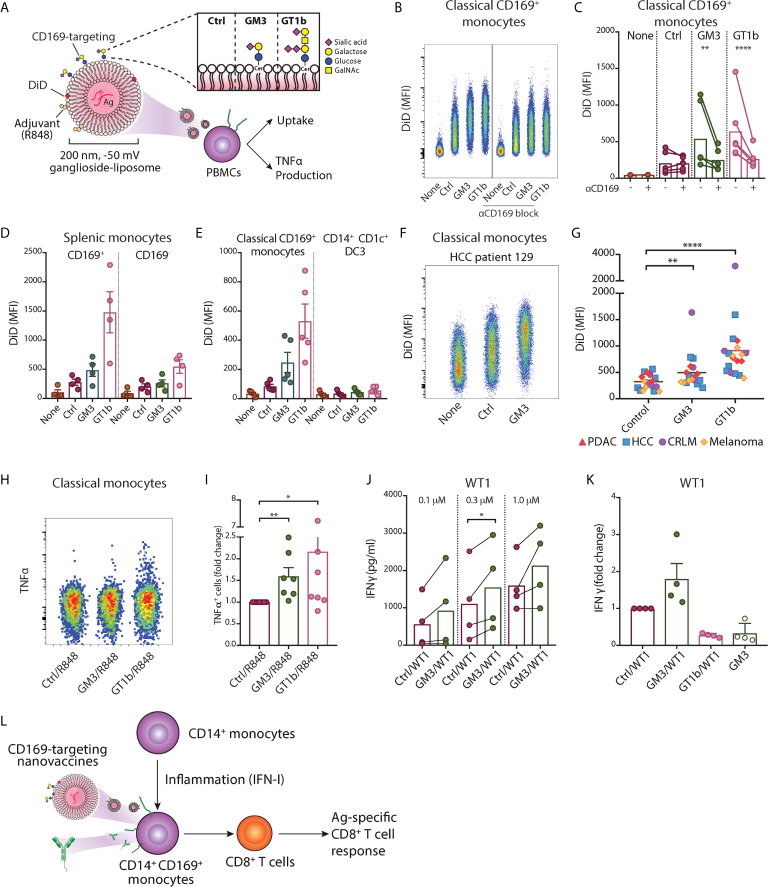
Ganglioside-liposomes deliver antigen/adjuvant to CD14^+^ CD169^+^ monocytes for presentation to CD8^+^ T cells. **(A)** Gangliosides GM3 and GT1b were incorporated into DiD-labeled liposomes and uptake was determined by flow cytometry. GalNAc, N-acetyl galactosamine; Cer, ceramide; Ctrl, control. Additionally, toll-like receptor 7/8 agonist R848 was incorporated and tumor-associated peptide was encapsulated. **(B, C)** Ganglioside liposome uptake by human CD14^+^ CD169^+^ monocytes as **(B)** representative plots and **(C)** quantification (*n* = 4) are shown. **(D)** Ganglioside-liposome uptake by human splenic autofluorescence (AF)^-^ CD14^+^ Lin(CD3/CD19/CD56)^–^ monocytes, repartitioned as CD169^+^ or CD169^-^ cells. **(E)** Reanalysis of ganglioside-liposome uptake on circulating classical CD169^+^ monocytes (HLA-DR^+^ Lin^-^ CD14^high^ CD1c^-^) and DC3 (HLA-DR^+^ Lin^-^ CD14^int^ CD1c^+^). **(F, G)** Ganglioside-liposome uptake by CD169^+^ classical monocytes of cancer patients as **(F)** representative plot and **(G)** quantification are shown. Friedman test using a two-stage linear step-up procedure of Benjamini, Krieger and Yekutieli, with Q = 0.05, was used. **adjusted P < 0.01, ****adjusted P < 0.0001. **(H, I)** PBMCs were incubated with R848-containing ganglioside-liposomes at 37°C for 45 min, washed, and cultured for five hours in complete medium, with the addition of brefeldin-A for the final three hours. TNFα production by classical monocytes was measured by intracellular flow cytometry, gated on live CD14^+^ CD16^-^ HLA-DR^+^ Lin(CD3/CD19/CD56)^–^ cells. **(H)** Representative plot from one donor and **(I)** quantification as fold change over control are shown. Data are mean ± SEM from 6-7 donors. **(J, K)** After CD14^+^ isolation, monocytes were incubated with different concentrations of Ganglioside/WT1/R848 liposome or control (Ctrl) liposome, washed, and WT1-specific CD8^+^ T cells were added. **(J)** IFNγ secretion after 24h was determined by ELISA. **(K)** Fold change of IFNγ secretion over Ctrl liposome is shown for GM3/WT1, GT1b/WT1, or GM3 devoid of peptide, at 1.0 µM dose. Paired t-tests were used. *P < 0.05, **P < 0.01, ****P < 0.001. **(L)** Two nanovaccine platforms, antibody- and liposome-based, deliver antigen to CD169+ monocytes for antigen-specific CD8^+^ T cell activation.

We previously described a nanovaccine platform targeting CD169^+^ cells using gangliosides, the endogenous ligands for CD169, and we showed that ganglioside-liposomes activated and delivered tumor antigens to Axl^+^ CD169^+^ DCs (20). These liposomes were 200 nm in size, negatively charged, and also contained antigen and adjuvant. We assessed their uptake in PBMCs and splenocytes using flow cytometry (**Table S1** and **Figure 5A** **)**. Within circulating classical CD169^+^ monocytes, we observed that inclusion of GM3 or GT1b as targeting moieties strongly increased the uptake of liposomes (**Figures 5B, C**). This uptake was significantly reduced when anti-CD169 antibody was used as blocking antibody prior to addition of liposomes, indicating that uptake was mediated primarily by ganglioside-CD169 interaction (**Figure 5C**). A similar uptake of ganglioside-liposomes was observed in splenic CD14^+^ CD169^+^ monocytes (**Figure 5D** and **Figure S5A**). Ganglioside-liposomes can thus be taken up by both, blood and splenic CD14^+^ CD169^+^ monocytes.

Furthermore, we performed reanalysis of our previous findings on ganglioside-liposome uptake by blood CD14^+^ cells to reevaluate possible ganglioside-liposome uptake by CD14-expressing DC3 (20). Since CD88 was not included in this panel, here we defined classical monocytes as CD14^high^ CD1c^-^ cells and CD14^dim^ CD1c^+^ as DC3 (**Figure S5B**). While CD14^high^ CD1c^-^ CD169^+^ classical monocytes took up ganglioside-liposomes, we did not detect significant ganglioside-liposome uptake by CD14^dim^ CD1c^+^ DC3 population (**Figure 5E**). However, upon DC3 repartition based on CD169 expression, we observed a similar trend on ganglioside-liposome uptake by CD169-expressing DC3s, albeit to a much lower extent than the CD169^+^ monocytes (**Figure S5C**). This suggests that among CD14-expressing cells, ganglioside liposomes are largely taken up by CD169^+^ classical monocytes rather than CD169^+^ DC3.

We then assessed whether the ganglioside-liposomes could target CD169^+^ monocytes in cancer patients. Similar to our findings in healthy individuals, both GM3- and GT1b-liposomes were taken up by CD169^+^ classical monocytes of all cancer patients we tested (**Figures 5F, G**), further supporting the potential of targeting CD169^+^ monocytes as a vaccination strategy.

Finally, we evaluated antigen/adjuvant delivery to CD169^+^ monocytes by ganglioside-liposomes. To determine the capacity of ganglioside-liposome to activate monocytes, we incorporated TLR7/8 agonist R848 into the liposomes. We showed that GM3/R848 and GT1b/R848 liposomes induced TNFα expression in total CD14^+^ monocytes, significantly higher than the non-targeting control/R848 liposome (**Figures 5H, I**). Next, we evaluated antigen presentation capacity of CD14^+^ CD169^+^ monocytes after antigen delivery by ganglioside-liposomes. We encapsulated pancreatic cancer-associated antigen WT1 short peptide into the liposomes, and incubated them with CD14^+^ isolated monocytes. Following co-culture with WT1-specific CD8^+^ T cell clone, we measured the amount of secreted IFNγ. Upon incubation with GM3/WT1/R848 liposome, CD14^+^ monocytes were able to stimulate higher secretion of IFNγ by WT1 CD8^+^ T cells as compared to control liposome (**Figures 5J, K**). Interestingly, although GT1b-liposome uptake was higher than GM3-liposome uptake, it did not lead to activation of WT1 CD8^+^ T cells. This data indicates that GM3-liposomes are able to target and deliver tumor antigen to CD14^+^ CD169^+^ monocytes leading to a strong CD8^+^ T cell stimulation.

## Discussion

CD169^+^ monocytes are detected under homeostatic and inflammatory conditions, however, their phenotype and functional role in T cell activation is underexplored. Here, we show that CD14^+^ CD169^+^ monocytes exhibit a higher activation phenotype with enhanced capacity for antigen presentation and CD8^+^ T cells activation. Our spectral cytometry data show that they are distinct from the recently defined CD14^+^ DC3 population, and that they are present in the circulation of healthy donors, SARS-CoV-2-infected patients, as well as patients with five different types of cancer. Furthermore, we show that CD169-targeting nanovaccines can deliver antigen/adjuvant to CD169^+^ monocytes that leads to robust antigen-specific T cell activation, indicating their potential for novel CD169 targeting vaccination strategies.

The increase of CD169 expression on monocytes was initially described in patients with increased IFN-I signature, such as systemic sclerosis (27). A high level of IFNα in the circulation of systemic lupus erythematosus patients was demonstrated to increase the capacity of monocytes to activate CD4^+^ T cells and to contribute to the break of tolerance (47). Furthermore, exposure of IFNα to monocytes was shown to lead to upregulation of maturation markers, and an increased potency of monocytes to activate CD4^+^ T cells in an allogeneic setting (48). This was confirmed in a more recent unbiased proteomic analysis, in which CD169 was among the highest upregulated membrane proteins by IFNα treatment, along with HLA-molecules and co-stimulatory markers (49). The enhanced capacity of T cell activation by IFN-treated monocytes is therefore attributed to the increased expression of HLA molecules and these activating ligands. In line with these findings, our data have demonstrated that IFNα-exposed monocytes showed increased HLA- and co-stimulatory molecules and had enhanced capacity to cross-present antigen to CD8^+^ T cells.

Although DC1 has the highest proficiency in cross-presentation of cell-associated antigens (50–53), monocytes and monocyte-derived cells are also capable to uptake exogenous antigen and process them for CD8^+^ T cell activation (48, 54–56). Here, we showed that IFNα-treated CD169^high^ monocytes were able to present both pre-processed and unprocessed gp100 peptides to antigen-specific CD8^+^ T cells. Interestingly, monocytes loaded with MART1 long peptide were shown to retain this peptide and to present it to CD8^+^ T cells after a full differentiation into moDC with GM-CSF/IL-4 (54). *Ex vivo* loading of monocytes with antigen followed by intravenous transfer of these loaded monocytes was shown to induce strong anti-tumor T cell responses in several mouse models (57). In this study, antigen transfer to DC1s by monocytes appeared to be involved. As we previously showed a similar collaboration between CD169^+^ macrophages and DC1 in mice (58), the potential interplay between CD169^+^ monocytes, DC, and CD8^+^ T cells in humans needs further investigation.

CD169^+^ monocytes have been proposed to be a diagnostic biomarker for viral infections including in COVID-19 patients with high sensitivity (59–61). Several studies have also revealed CD169 alterations when comparing mild and severe COVID-19 cases. CD169-expressing monocytes were found to be more prominent in mild cases and the amount of CD169 expression correlated with plasma IFNα levels (60, 62). CD169 also identified early activated monocyte clusters in COVID-19 patients, that were absent in healthy controls (63), reminiscent to our scRNA-seq analysis. In this study, CD169 trajectory analysis predicted that CD169^+^ monocytes could be derived from both, classical and intermediate monocytes, thus corroborating our analysis. Additionally, we observed a higher proportion of CD169^+^ intermediate monocytes as compared to classical or non-classical subsets in COVID-19 patients. Intermediate monocytes are known to be increased during infection, including SARS-CoV-2 (64), and it is likely that these CD169^+^ intermediate monocytes were transitioning from activated classical monocytes. Remarkably, the frequency of CD169^+^ monocytes was also associated with high IFNγ levels (63). These data suggests that CD169^+^ monocytes reflect high IFN levels and may potentially be involved in the activation of T cell responses required for virus clearance in COVID-19 patients.

In cancer, monocytes are known to play dual roles in promoting or suppressing tumor growth (11). CD169^+^ monocytes were previously reported to be highly increased in the circulation of patients with colorectal carcinoma, to produce high amount of IL-10, and were associated with tumor-infiltrating CD169^+^ monocytes/macrophages and poor prognosis (65). In breast cancer, CD169 also marked tumor-associated macrophages and *SIGLEC1* expression was associated with poor outcome (66). On the other hand, *SIGLEC1* expression was correlated with immune cell infiltration in endometrial cancer and survival (67). Along the same line, tumor-infiltrating CD169^+^ monocytes/macrophages were shown to be a good prognostic marker in hepatocellular carcinoma (68). In hepatocellular carcinoma, tumor-infiltrating CD169^+^ monocytes/macrophages presence was correlated with CD8^+^ T cell frequency and both were found in close proximity. Moreover, hepatocellular carcinoma-associated CD169^+^ monocytes/macrophages showed elevated expression of HLA-DR and CD86, similar to the pattern we observed in CD169^+^ monocytes. Furthermore, several studies have reported that the presence of CD169^+^ macrophages in the tumor draining lymph node is associated with better prognosis in multiple types of cancer (42–44). Thus, the role of CD169-expressing monocytes/macrophages is highly dependent on tumor types and tissues investigated.

Interestingly, CD169 molecule itself has been suggested to promote T cell activation. Addition of recombinant human CD169 to anti-CD3-stimulated PBMCs induced CD8^+^ T cell proliferation and cytokine production in an autologous condition (68). In an allogeneic setting, blocking of CD169 expressed on IFN-α treated monocytes, led to a reduced proliferation and cytokine production by CD8^+^ and CD4^+^ T cells (69). However, also contradictory data exists, in which CD169 is involved in T cell suppression (70, 71). Further research into the interaction of CD169 with T cells and to elucidate the ligands involved will be necessary. These studies suggest that CD169 expression on monocytes could potentially act as an additional signal that can influence CD8^+^ T cell activation.

Finally, to evaluate the potential of CD169^+^ monocytes for a vaccination strategy, we used two forms of CD169-targeting nanovaccine platforms (**Figure 5L**). Our data showed that anti-CD169 Ab-Ag conjugate and ganglioside-liposomes were efficiently taken up by CD169^+^ monocytes and delivered tumor-associated antigens which led to robust antigen-specific CD8^+^ T cell responses. This is in line with our previous reports using CD169^+^ moDC and Axl^+^ DC (20, 37). Interestingly, only GM3-liposome, and not GT1b-liposome, that was able to deliver antigen for CD8^+^ T cells activation by CD169^+^ monocytes, similar to Axl^+^ DCs. Future experiments investigating ganglioside-liposome intracellular trafficking, peptide processing and presentation on MHC are needed to determine the underlying mechanisms. Since CD169 is also expressed by Axl^+^ DC, targeting the CD169 molecule would give additional benefits of targeting both Axl^+^ DCs to prime naïve CD8^+^ T cells and CD169^+^ monocytes to stimulate antigen-experienced T cells. In addition, intravenous administration of CD169-targeting nanovaccines in mice specifically targets splenic CD169^+^ macrophages, leading to strong T cell responses that depend on DC1 (34, 35, 37, 72). A similar type of macrophage is present in human spleen and are described as perifollicular macrophages (37), but have not been studied because of their scarcity. Together, this suggest that cancer vaccines targeting CD169 could potentially mediate T cell activation *via* the consorted action of targeting splenic CD169^+^ perifollicular macrophages, Axl^+^ DCs, and CD169^+^ monocytes.

Taken together, our data show that CD169^+^ monocytes are activated monocytes that are present in both, healthy and diseased conditions, including in viral infections and cancer. Increased expression of CD169 in monocytes is driven by IFN-I and it is accompanied by increased expression of co-stimulatory and HLA molecules. CD169^+^ monocytes exhibit an enhanced antigen presentation and CD8^+^ T cell activation capacity, and can be selectively targeted and activated by CD169-targeting nanovaccines. Thereby, CD169^+^ monocytes are APCs with potential for an effective targeted nano-vaccination strategy.

## Data Availability Statement

The raw data supporting the conclusions of this article will be made available by the authors, without undue reservation.

## Ethics Statement

The studies involving human participants were reviewed and approved by Medical Ethical Committee, Review Committee Biobank, Amsterdam UMC. The patients/participants provided their written informed consent to participate in this study.

## Author Contributions

AA and JH conceived and planned the experiments. AA wrote the first draft of the manuscript. AA, KO, JG, MN, ER, AS, EZ, EN, HK, and MK carried out the experiments, analyzed, and interpreted the data. EZ, GK, TG, AE, AS, EN, and JG-V provided clinical materials and information. JS, JG-V, TG, GS, YK, and JH supervised the study. All authors contributed to the article and approved the submitted version.

## Funding

This work was supported by grants from the Dutch Cancer Society (VU2016-10449, VU2019-12802) to JH, YK, TG, and (VU2019-12802) to AE, from the Phospholipid Research Center to JH and YK (JDH-2020-082/1-1), from NWO ZonMW (TOP 91218024) to JH and GS, and de Bennink and Cancer Center Amsterdam Foundations to GK.

## Conflict of Interest

The authors declare that the research was conducted in the absence of any commercial or financial relationships that could be construed as a potential conflict of interest.

## Publisher’s Note

All claims expressed in this article are solely those of the authors and do not necessarily represent those of their affiliated organizations, or those of the publisher, the editors and the reviewers. Any product that may be evaluated in this article, or claim that may be made by its manufacturer, is not guaranteed or endorsed by the publisher.
