# Supplementary material for: CD169 Defines Activated CD14+ Monocytes With Enhanced CD8+ T Cell Activation Capacity
Source: Front Immunol. 2021 Jul 28;12:697840. doi: 10.3389/fimmu.2021.697840 (PMC8356644; doi:10.3389/fimmu.2021.697840)
Supplement: Supplementary file 1 [file DataSheet_1.pdf]

Supplementary Material

Supplementary Figures & Tables

1 Supplementary Figures

Figure S1

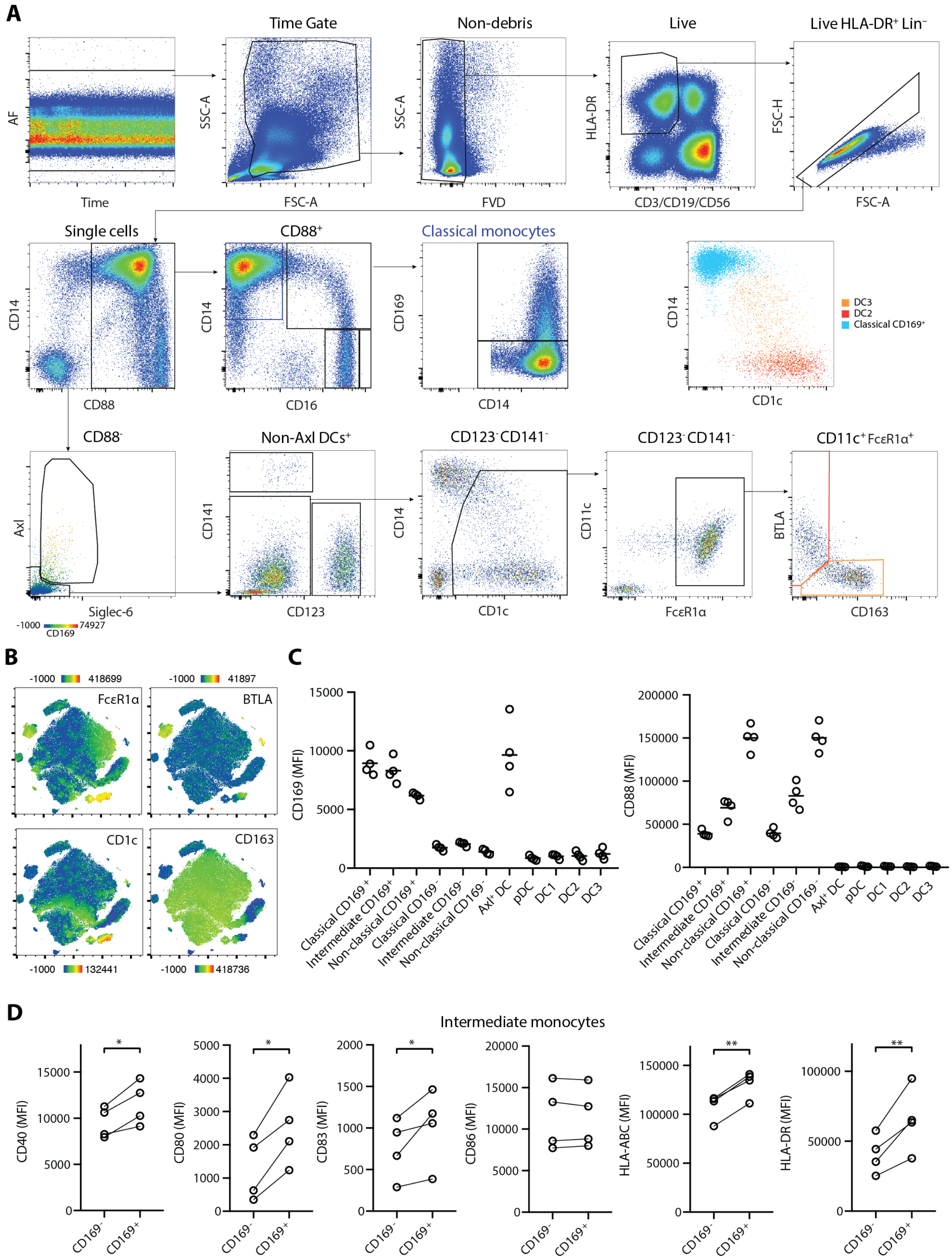

Figure S1. CD14$^+$ CD169$^+$ monocytes display enhanced maturation status. (**A**) Gating strategy to define CD88$^+$ monocytes and CD88$^-$ dendritic cells (DCs) populations within HLA-DR$^+$ Lin(CD3/CD19/CD56)$^-$ cells. Monocytes are repartitioned as classical (CD14$^+$ CD16$^-$), intermediate (CD14$^+$ CD16$^+$), and non-classical (CD14$^-$ CD16$^+$) monocytes. The DC populations comprise of Axl$^+$ DC (Axl$^+$ Siglec-6$^+$), plasmacytoid DC (pDC, CD123$^+$), DC1 (CD141$^+$), DC2 (CD1c$^+$ BTLA$^+$ CD163$^-$), and DC3 (CD1c$^+$ BTLA$^-$ CD163$^+$). DC3 is distinguishable from monocytes by CD88$^-$ FcεRIα$^+$ expression. (**B**) The expression of FcεR1α, CD1c, BTLA, and CD163 on tSNE plots. (**C**) Expression of CD169 and CD88 in monocytes and DC subsets (*n* = 4). (**D**) Expression of maturation markers comparing CD169$^+$ and CD169$^-$ intermediate monocytes (*n* = 4). Paired t-tests were used. * $P < 0.05$, ** $P < 0.01$.

Figure S2

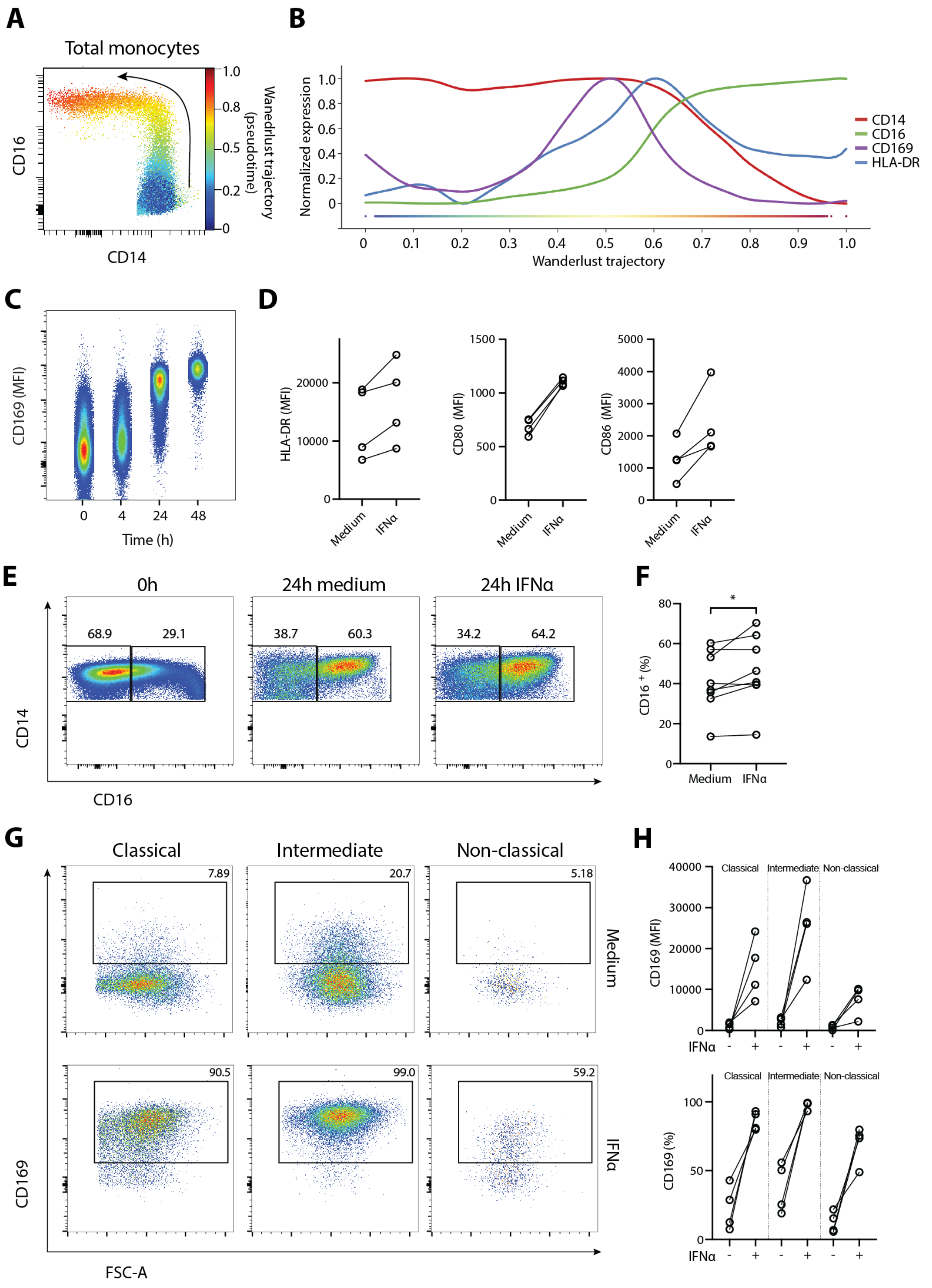
A
Total monocytes
CD16
CD14
Wanderlust trajectory (pseudotime)
1.0
0.8
0.5
0.2
0
B
Normalized expression
Wanderlust trajectory
CD14
CD16
CD169
HLA-DR
C
CD169 (MFI)
Time (h)
0
4
24
48
D
HLA-DR (MFI)
CD80 (MFI)
CD86 (MFI)
Medium
IFNα
E
0h
24h medium
24h IFNα
68.9
29.1
38.7
60.3
34.2
64.2
CD14
CD16
F
CD16 + (%)
*
Medium
IFNα
G
Classical
Intermediate
Non-classical
7.89
20.7
5.18
90.5
99.0
59.2
Medium
IFNα
CD169
FSC-A
H
CD169 (MFI)
CD169 (%)
Classical
Intermediate
Non-classical
IFNα

Figure S2

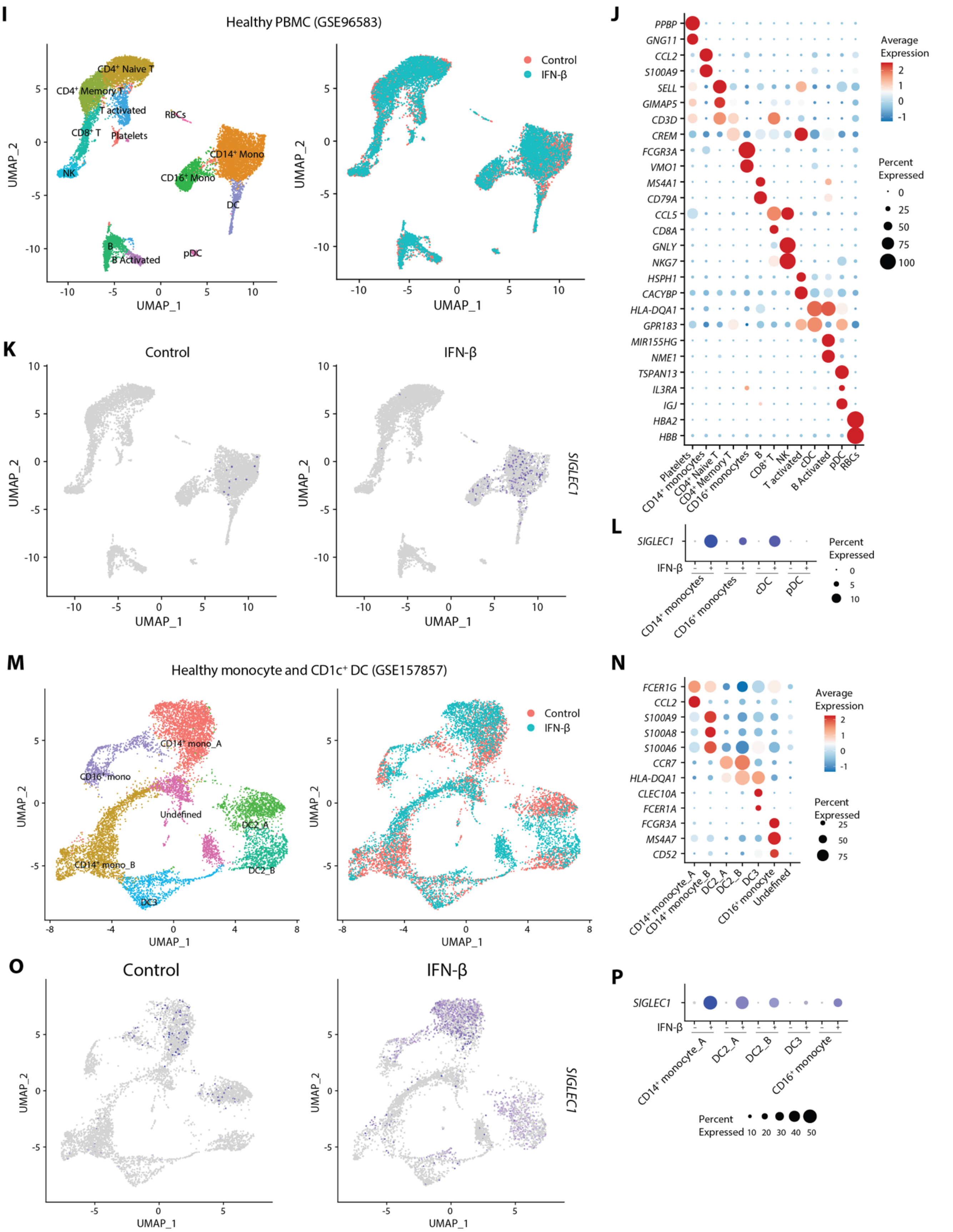
I
Healthy PBMC (GSE96583)
CD4+ Naive T
CD4+ Memory T
T activated
RBCs
CD8+ T
Platelets
CD14+ Mono
NK
CD16+ Mono
DC
B
B Activated
pDC
Control
IFN-β
UMAP_1
UMAP_2
J
Average Expression
Percent Expressed
PPBP
GNG11
CCL2
S100A9
SELL
GIMAP5
CD3D
CREM
FCGR3A
VMO1
MS4A1
CD79A
CCL5
CD8A
GNLY
NKG7
HSPH1
CACYBP
HLA-DQA1
GPR183
MIR155HG
NME1
TSPAN13
IL3RA
IGJ
HBA2
HBB
Platelets
CD14+ monocytes
CD4+ Naive T
CD4+ Memory T
CD16+ monocytes
B
CD8+ T
NK
T activated
cDC
B Activated
pDC
RBCs
K
Control
IFN-β
SIGLEC1
L
SIGLEC1
IFN-β
CD14+ monocytes
CD16+ monocytes
cDC
pDC
M
Healthy monocyte and CD1c+ DC (GSE157857)
CD14+ mono_A
CD16+ mono
Undefined
DC2_A
CD14+ mono_B
DC2_B
DC3
N
FCER1G
CCL2
S100A9
S100A8
S100A6
CCR7
HLA-DQA1
CLEC10A
FCER1A
FCGR3A
MS4A7
CD52
CD14+ monocyte_A
CD14+ monocyte_B
DC2_A
DC2_B
DC3
CD16+ monocyte
Undefined
O
Control
IFN-β
SIGLEC1
P
SIGLEC1
IFN-β
CD14+ monocyte_A
DC2_A
DC2_B
DC3
CD16+ monocyte
Percent Expressed 10 20 30 40 50

Figure S2. CD169 expression in monocytes is driven by IFN-I. (**A,B**) Wanderlust trajectory analysis of monocyte population using $CD14^+$ $CD169^-$ monocytes as starting population overlaid by conventional gating of monocyte subsets. $CD14^+$ $CD169^-$ cells were used for input and the following markers were used: CD163, CD14, CD11c, HLA-DR, CD16, CD169, CD141, CD88, and CD1c. (**A**) Example of development trajectory of classical to intermediate to non-classical monocyte populations. (**B**) Wanderlust trajectory plots showing normalized expression of CD14, CD16, CD169, and HLA-DR. (**C**) $CD14^+$ monocytes were isolated and treated with 1,000 IU/ml IFNα for the indicated time-points. Representative plots showing CD169 expression are shown. (**D**) Expression of HLA-DR, CD80, and CD86 of $CD14^+$ monocytes treated with medium or 1,000 IU/ml IFNα for 24h (n = 4). (**E**,**F**) CD16 expression within $CD14^+$ monocytes of untreated, medium-treated, and IFNα-treated shown as (**E**) representative plots and (**F**) quantification (n = 8). Paired t-test, * $P < 0.05$. (**G**,**H**) Monocytes were isolated using percoll density gradient and cultured with medium or 1,000 IU/ml IFNα. Expression of CD169 on classical ($CD14^+$ $CD16^-$), intermediate ($CD14^+$ $CD16^+$), and non-classical ($CD14^-$ $CD16^+$) monocytes is shown as (**G**) representative plots and (**H**) quantification (n = 4). (**I-L**) Analysis of public sc-RNAseq dataset of PBMCs from healthy controls treated with or without IFNβ for 6 h (GSE96583) [1] using Seurat Integration pipeline [2]. pDC, plasmacytoid DC; cDC, conventional DC; RBC, red blood cells. (**I**) UMAP analysis showing distribution of control and stimulated cells across clusters. (**J**) Dotplot showing gene expression of known lineage markers across clusters. (**K**,**L**) Expression of *SIGLEC1* on control or IFNβ-treated cells shown as (**K**) UMAP analysis and (**L**) dotplot. (**M**-**P**) Analysis of public sc-RNAseq dataset of isolated monocytes and $CD1c^+$ DCs from healthy controls treated with or without IFNβ for 18 h (GSE157857) [3]. (**M**) UMAP analysis showing distribution of control and stimulated cells across clusters. (**N**) Dotplot showing gene expression of known lineage markers across clusters. (**O**,**P**) Expression of *SIGLEC1* on control or IFNβ-treated cells shown as (**O**) UMAP analysis and (**P**) dotplot.

Figure S3

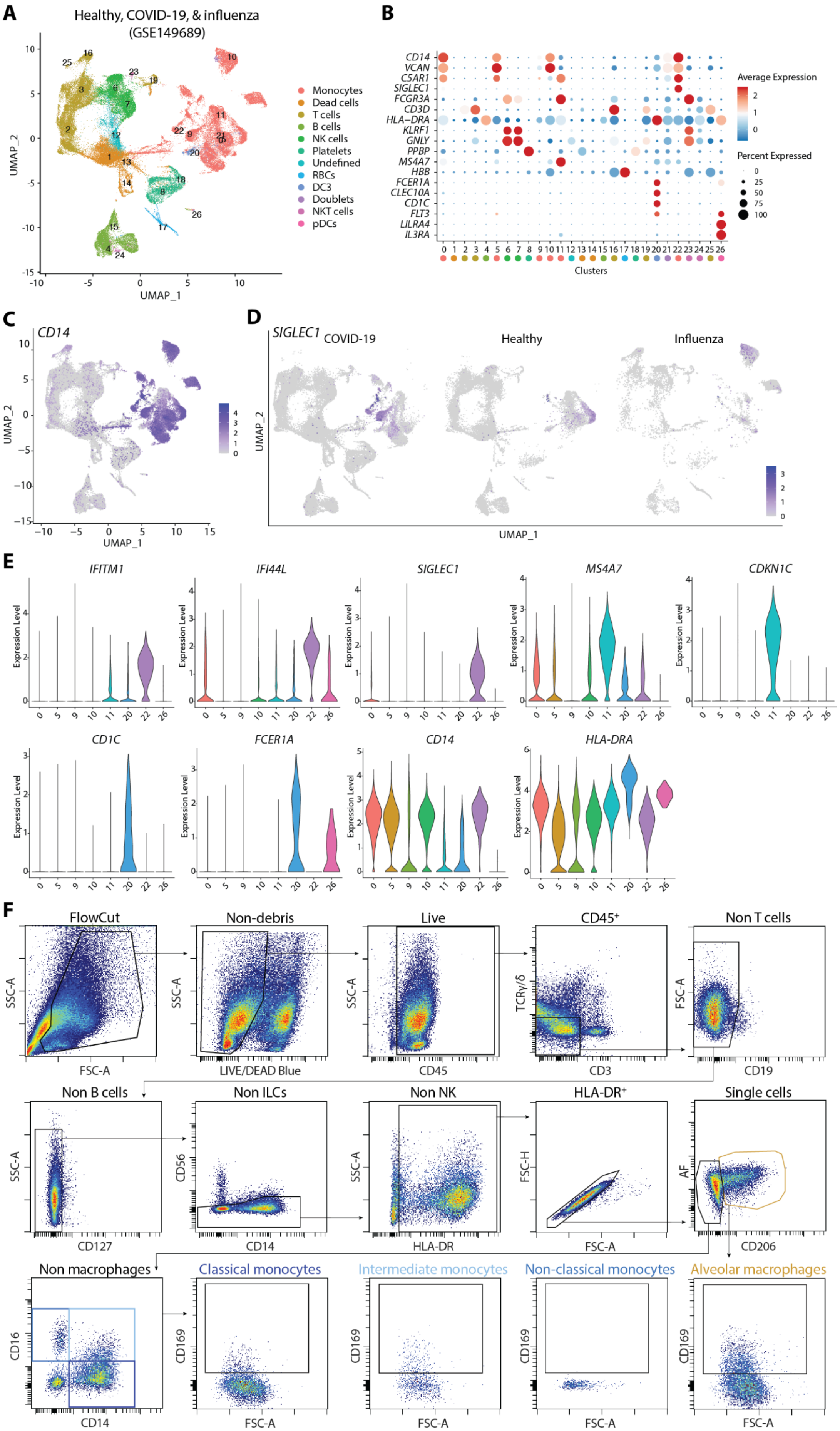
A
Healthy, COVID-19, & influenza (GSE149689)
UMAP_1
UMAP_2
Monocytes
Dead cells
T cells
B cells
NK cells
Platelets
Undefined
RBCs
DC3
Doublets
NKT cells
pDCs
B
CD14
VCAN
C5AR1
SIGLEC1
FCGR3A
CD3D
HLA−DRA
KLRF1
GNLY
PPBP
MS4A7
HBB
FCER1A
CLEC10A
CD1C
FLT3
LILRA4
IL3RA
Clusters
Average Expression
Percent Expressed
C
CD14
D
SIGLEC1
COVID-19
Healthy
Influenza
E
IFITM1
IFI44L
SIGLEC1
MS4A7
CDKN1C
CD1C
FCER1A
CD14
HLA-DRA
Expression Level
F
FlowCut
Non-debris
Live
CD45+
Non T cells
SSC-A
FSC-A
LIVE/DEAD Blue
CD45
TCRγ/δ
CD3
CD19
Non B cells
Non ILCs
Non NK
HLA-DR+
Single cells
CD127
CD56
CD14
HLA-DR
FSC-H
AF
CD206
Non macrophages
Classical monocytes
Intermediate monocytes
Non-classical monocytes
Alveolar macrophages
CD16
CD169

Figure S3. $CD14^+$ $CD169^+$ monocytes are present in COVID-19 patients and exhibit activated phenotype. (**A**) Analysis of public sc-RNAseq dataset of PBMCs from patients with COVID-19 (n = 9), severe influenza (n = 5) and healthy controls (n = 4) using Seurat pipeline and projected onto UMAP. (**B**) Dotplot showing gene expression of known lineage markers across clusters. (**C**) UMAP analysis showing *CD14* expression in all groups. (**D**) UMAP analysis showing *SIGLEC1* expression in different groups. (**E**) Violin plots of selected genes (*IFITM1, IFI44L, SIGLEC1*) defining cluster 22 (see **Table S3**), non-classical monocytes markers *MS4A7* and *CDKN1C*, DC3 markers *CD1C* and *FCER1A* in monocytes and DC clusters, *CD14*, and *HLA-DRA*. (**F**) Gating strategy to define $CD169^+$ monocytes or macrophages within $HLA\text{-}DR^+$ $Lin(CD3/CD19/CD56/CD127)^-$ cells of bronchoalveolar lavage fluid from COVID-19 patients adapted from Saris et al [4]. Alveolar macrophages are defined as autofluorescence $(AF)^{high}$ $CD206^+$ Lin^- cells. $CD206^-$ cells are further repartitioned based on CD14 and CD16 to identify classical ($CD14^+$ $CD16^-$), intermediate ($CD14^+$ $CD16^+$), and non-classical monocytes ($CD14^-$ $CD16^+$).

Figure S4

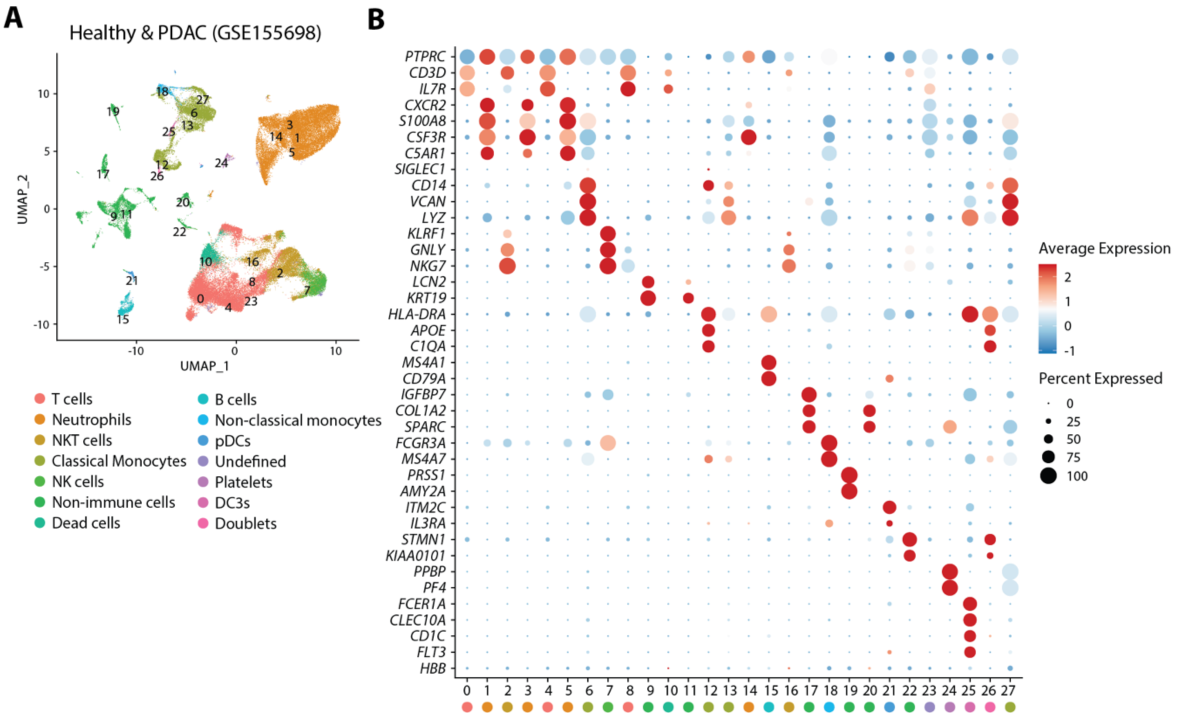

Figure S4. Analysis of scRNA-seq in PDAC patients. (**A**) Analysis of public sc-RNAseq dataset of PBMCs from PDAC (n = 12) and healthy controls (n = 4) using Seurat algorithm and projected onto UMAP space where cell clusters are indicated. (**B**) Dotplot showing gene expression of known lineage markers across clusters.

Figure S5

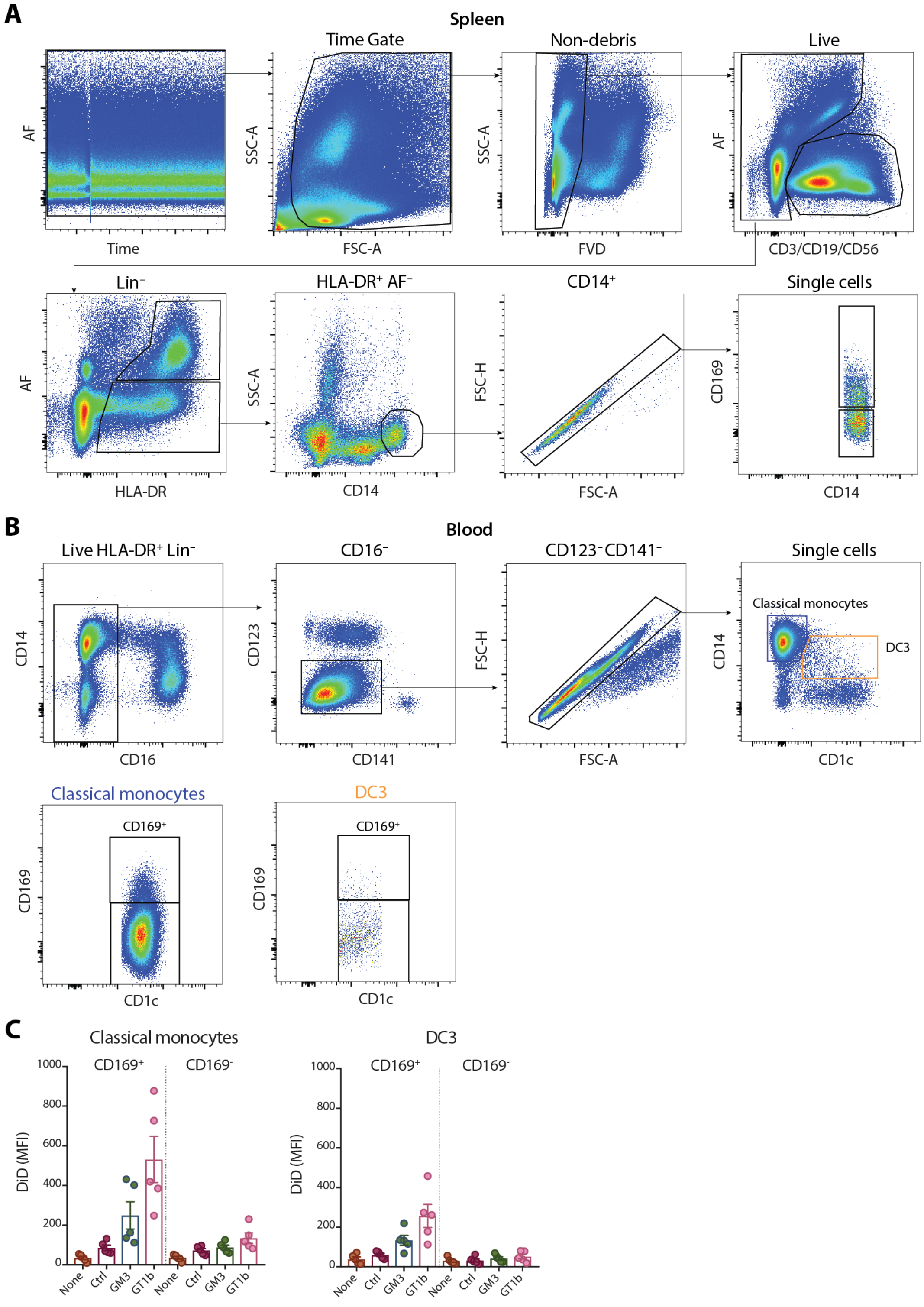

Figure S5. CD14^{+} CD169^{+} monocytes uptake of ganglioside-liposomes. (**A**) Gating strategy to define CD14^{+} monocytes within HLA-DR^{+} Lin(CD3/CD19/CD56)$^{-}$ cells from digested human spleen. Macrophages were excluded based on autofluorescence (AF). (**B**) Reanalysis of our published data [5] redefining classical monocytes (CD14^{high} CD1c^{-}) and DC3 (CD14^{int} CD1c^{+}), and their CD169

expression. (**C**) Ganglioside-liposome uptake comparing CD169$^+$ and CD169$^-$ fractions of classical monocytes and DC3 populations (*n* = 4).

2 Supplementary Tables

2.1 Table S1

	Size (nm, mean ± SD)	Polydispersity index (mean ± SD)	Zeta potential (mean ± SD)
Ctrl/R848/WT1	192.9 ± 0.1	0.07 ± 0.01	-53.53 ± 3.3
GM3/R848/WT1	206.1 ± 0.1	0.07 ± 0.05	-51.37 ± 6.9

Physical properties of liposomes determined by dynamic light scattering.

2.2 Table S2

Antigen	Label	Clone	Company	Catalog #
CD163	BV421	GHI/61	Biolegend	333612
CD83	BV421	HB15e	Biolegend	305323
CD123	BV510	6H6	Biolegend	302046
HLA-ABC	BV510	W6/32	Biolegend	311435
CD14	BV605	M5E2	Biolegend	301834
CD11c	BV650	B-ly6	BD	563403
HLA-DR	BV711	L243	Biolegend	307644
TNF-a	BV750	MAb11	BD	566359
CD16	BV786	3G8	Biolegend	302045
CD40	BV785	5C3	Biolegend	334339
AXL	AF488	108724R	R&D	FAB154RG-100UG
CD86	FITC	BU63	Immunotools	21480863
CD169	PE	7-239	BD	565248
CD88	PE/Dazzle594	S5/1	Biolegend	344318
CD3	PE-Cy5	UCHT1	BD	561007
CD19	PE-Cy5	HIB19	BD	561904
CD56	PE-Cy5	B159	BD	560993
CD1c	PerCP-ef710	L161	eBioscience	46-0015-42
CD141	PE-Cy7	M80	Biolegend	344109
CD80	PE-Cy7	2D10	Biolegend	305217
BTLA	APC	MIH26	Biolegend	344509
CD16	APC	LNK16	Immunotools	21279166
SIGLEC-6	AF700	767329	R&D	FAB2859RN
FcεRIα	APC/Fire750	AER-37 (CRA-1)	Biolegend	334643

List of antibodies used in this study.

2.3 Table S3

Gene	p_val	avg_log2FC	pct.1	pct.2	p_val_adj
IFITM1	0	1.76245363	0.871	0.149	0
IFI44L	0	1.70910688	0.945	0.285	0
OTOF	0	1.06278481	0.454	0.03	0
SIGLEC1	0	1.02993838	0.722	0.132	0
RSAD2	0	1.01997263	0.63	0.075	0
USP18	0	0.55124055	0.409	0.031	0
ISG15	3.57E-289	2.46901138	0.984	0.497	8.31E-285
ANKRD22	2.44E-284	0.86885427	0.515	0.072	5.70E-280
C15orf48	2.23E-271	1.7005155	0.658	0.126	5.20E-267
HERC5	1.72E-265	0.91945116	0.628	0.115	4.00E-261
IFI6	1.69E-259	2.06458774	0.984	0.545	3.94E-255
XAF1	1.49E-243	1.56898991	0.947	0.414	3.48E-239
MX1	3.15E-238	1.44699096	0.9	0.333	7.34E-234
APOBEC3A	9.71E-235	1.94481898	0.955	0.424	2.26E-230
PTX3	1.28E-232	1.21868209	0.583	0.113	2.98E-228
EPSTI1	3.05E-232	1.38666151	0.9	0.36	7.12E-228
IFITM3	8.97E-231	1.84335761	0.992	0.775	2.09E-226
SERPING1	3.93E-219	0.85258214	0.624	0.133	9.16E-215
CCL2	3.47E-218	1.25825614	0.264	0.024	8.08E-214
MX2	1.13E-214	1.32924094	0.922	0.397	2.63E-210
OASL	2.04E-213	0.95242384	0.716	0.187	4.75E-209
HCAR3	1.82E-199	0.70591962	0.393	0.058	4.23E-195
IFI27	3.71E-195	2.76641143	0.652	0.177	8.64E-191
ATF3	1.33E-193	1.10643691	0.585	0.137	3.10E-189
ABCA1	1.47E-189	0.74351951	0.599	0.136	3.42E-185
G0S2	9.10E-188	1.9761087	0.904	0.364	2.12E-183
AC020656.1	2.65E-185	1.50949139	0.998	0.837	6.18E-181
ETV7	9.76E-184	0.40037119	0.329	0.043	2.27E-179
ISG20	2.11E-183	1.07329564	0.673	0.186	4.91E-179
OAS3	4.38E-181	0.86586041	0.712	0.21	1.02E-176
LY6E	7.55E-181	1.2838889	0.984	0.658	1.76E-176
NEXN	3.24E-179	0.56016887	0.417	0.071	7.56E-175
IFIT3	5.54E-178	0.84660231	0.673	0.184	1.29E-173
IFIT1	2.34E-176	0.47314567	0.399	0.065	5.45E-172
HCAR2	2.67E-176	0.40836646	0.262	0.029	6.23E-172
IRF7	5.81E-176	1.08535135	0.847	0.359	1.35E-171
SPATS2L	9.36E-175	0.64709452	0.479	0.097	2.18E-170
IFITM2	1.41E-171	1.12994939	0.996	0.806	3.28E-167
FFAR2	2.01E-171	0.99258738	0.648	0.175	4.68E-167
PHLDA2	9.14E-171	0.84104677	0.562	0.134	2.13E-166
MAFF	1.55E-165	0.6695028	0.579	0.142	3.61E-161
PLAC8	1.83E-164	1.48473111	0.953	0.588	4.26E-160
ABCG1	2.94E-163	0.40952633	0.321	0.046	6.86E-159
MARCKS	1.14E-162	1.40546011	0.994	0.684	2.66E-158

GBP1	2.35E-161	1.13335781	0.867	0.361	5.48E-157
FCGR1B	9.64E-160	0.86468437	0.677	0.209	2.25E-155
PLEK	4.43E-157	1.17822648	0.992	0.788	1.03E-152
BCL2A1	1.11E-155	1.36652511	0.951	0.552	2.59E-151
SAMD9L	9.65E-153	0.80477496	0.687	0.216	2.25E-148
CARD16	1.48E-149	1.10593909	0.973	0.707	3.45E-145

List of top 50 genes of cluster 22 as compared to other monocyte populations (cluster 0, 5, 9, and 10) obtained using Seurat FindAllMarkers function. avg_log2FC = average log2 fold change. The percentages of cells where the gene is detected in cluster 22, and where the gene is detected on average in the other clusters, are shown as pct.1 and pct.2, respectively. p_val_adj = adjusted P value based on Bonferroni's correction.

3 References

[1] H.M. Kang, M. Subramaniam, S. Targ, M. Nguyen, L. Maliskova, E. McCarthy, E. Wan, S. Wong, L. Byrnes, C.M. Lanata, R.E. Gate, S. Mostafavi, A. Marson, N. Zaitlen, L.A. Criswell, and C.J. Ye, Multiplexed droplet single-cell RNA-sequencing using natural genetic variation. Nat Biotechnol 36 (2018) 89-94.

[2] T. Stuart, A. Butler, P. Hoffman, C. Hafemeister, E. Papalexi, W.M. Mauck, 3rd, Y. Hao, M. Stoeckius, P. Smibert, and R. Satija, Comprehensive Integration of Single-Cell Data. Cell 177 (2019) 1888-1902 e21.

[3] M. Girard, J.C. Law, M.I. Edilova, and T.H. Watts, Type I interferons drive the maturation of human DC3s with a distinct costimulatory profile characterized by high GITRL. Sci Immunol 5 (2020).

[4] A. Saris, T.D. Reijnders, E.J. Nossent, A.R. Schuurman, J. Verhoeff, S.V. Asten, H. Bontkes, S. Blok, J. Duitman, H.J. Bogaard, L. Heunks, R. Lutter, T. van der Poll, J.J. Garcia Vallejo, D.c. Art, and U.M.C.C.s.g. the Amsterdam, Distinct cellular immune profiles in the airways and blood of critically ill patients with COVID-19. Thorax (2021).

[5] A.J. Affandi, J. Grabowska, K. Olesek, M. Lopez Venegas, A. Barbaria, E. Rodriguez, P.P.G. Mulder, H.J. Pijffers, M. Ambrosini, H. Kalay, T. O'Toole, E.S. Zwart, G. Kazemier, K. Nazmi, F.J. Bikker, J. Stockl, A.J.M. van den Eertwegh, T.D. de Gruijl, G. Storm, Y. van Kooyk, and J.M.M. den Haan, Selective tumor antigen vaccine delivery to human CD169(+) antigen-presenting cells using ganglioside-liposomes. Proc Natl Acad Sci U S A 117 (2020) 27528-27539.
